# Remote-access thyroidectomy with the da Vinci SP system: feasibility in a cadaveric model

**DOI:** 10.3389/fsurg.2023.1196021

**Published:** 2023-06-15

**Authors:** Hubert Stein, Sang Wook Kang, Seung Young Heo, Markus Rheinwald

**Affiliations:** ^1^ Department of Surgical Applications Engineering, Intuitive Surgical Inc., Sunnyvale, CA, United States; ^2^ Department of Surgery, Yonsei University College of Medicine, Seoul, Republic of Korea

**Keywords:** single port, remote-access, thyroidectomy, presternal, submental, robotic

## Abstract

**Background:**

This study aims to study the feasibility of a remote-access thyroidectomy through presternal and submental approaches with the da Vinci SP system.

**Methods:**

Bilateral thyroidectomies were performed in five cadaveric models. A single incision in the presternal area was used in two cadavers, and a submental facelift incision approach was used in three cadavers.

**Results:**

Performing remote-access thyroidectomy was completed with a presternal approach in one cadaver and with the submental approach in three cadavers. The required skin flap development was minimal, and the docking time for the SP system was quick for all procedures. Time to full exposure of the thyroid gland after skin incision was less than 30 min for the presternal approach and less than 27 min for the submental procedure. Completing total thyroidectomies took 83 min in the presternal approach and between 67 and 127 min in the submental access. No additional ports were required to expose the gland and complete the bilateral resection.

**Conclusions:**

Total thyroidectomy was feasible with the da Vinci SP system in single incision presternal and submental approaches comparing promisingly with other currently applied robotic methods. Further studies will be required to assess whether a presternal or submental thyroidectomy with the da Vinci SP system provides clinical benefits in real patients.

## Introduction

Different approaches of robotic-assisted remote-access thyroidectomy with multiport da Vinci systems have been developed in the past 14 years since Chung performed the first such transaxillary (TA) approach in October 2007 (1). In addition to the transaxillary route, surgeons have applied the bilateral axillobreast (BABA) (2, 3), retroauricular or facelift (FL) (4, 5), and transoral (TORT) (6, 7) approaches safely in patients. Nevertheless, the method that produces the best clinical outcomes is not yet certain, as each robotic-assisted thyroidectomy approach has its advantages and disadvantages. There are still concerns regarding various aspects of the procedure such as the operative invasiveness to create the subcutaneous flaps accessing the gland, gasless versus gas, difficulty to reach the contralateral side for bilateral dissections, distance from skin incision to the gland, location and cosmesis of the skin incision, surgical view onto the anatomy, ability to use all robotic arms, need for retractors, nerve injuries, infections, and control of hemorrhage, among others (8). We applied a presternal approach to assess whether any of these aspects could be overcome with the next-generation single-port da Vinci SP system. The da Vinci SP system is a progression from the da Vinci multiport systems whereby three instruments and a stereoscopic camera are employed through a single port on a single instrument arm (Entry Guide Manipulator, EGM). This EGM is docked to the SP cannula of 28 mm outer diameter placed in the body wall. Elbow joints proximal to the wrist on the instruments, camera triangulation, and independent planar movements of the instrument tips allow access to narrow workspaces and avoid external collisions. Initial clinical cases with the da Vinci SP system in thyroidectomy were limited to the transaxillary approach but have shown excellent cosmetic outcomes and reduced postoperative pain due to a smaller skin incision and the reduced extent of flap dissection (9). The aim of this study was to evaluate the feasibility of a remote-access thyroidectomy through presternal and submental approaches with the da Vinci SP system and allow for a basic initial comparison to the other robotic thyroidectomy approaches.

## Materials and methods

The procedures were performed in five human cadavers in a surgical lab certified for research and development (DIN EN ISO 9001:2015) and carried out in accordance with ethical standards and the declaration of Helsinki 1964 and its subsequent updates. Further, it did not require approval from the institutional review board as living humans were not included. Table 1 lists the characteristics of the cadaveric models used and the approach applied.

**Table 1:** Model characteristics.

Cadaver no.	Approach	Gender	Age (years)	Height (cm)	Weight (kg)	BMI
1	Presternal	F	49	158	47	19
2	Presternal	F	96	163	61	23
3	Submental	F	82	153	40	17
4	Submental	M	74	175	45	15
5	Submental	M	65	176	82	26.5

### Presternal thyroidectomy

**Figure 1:**
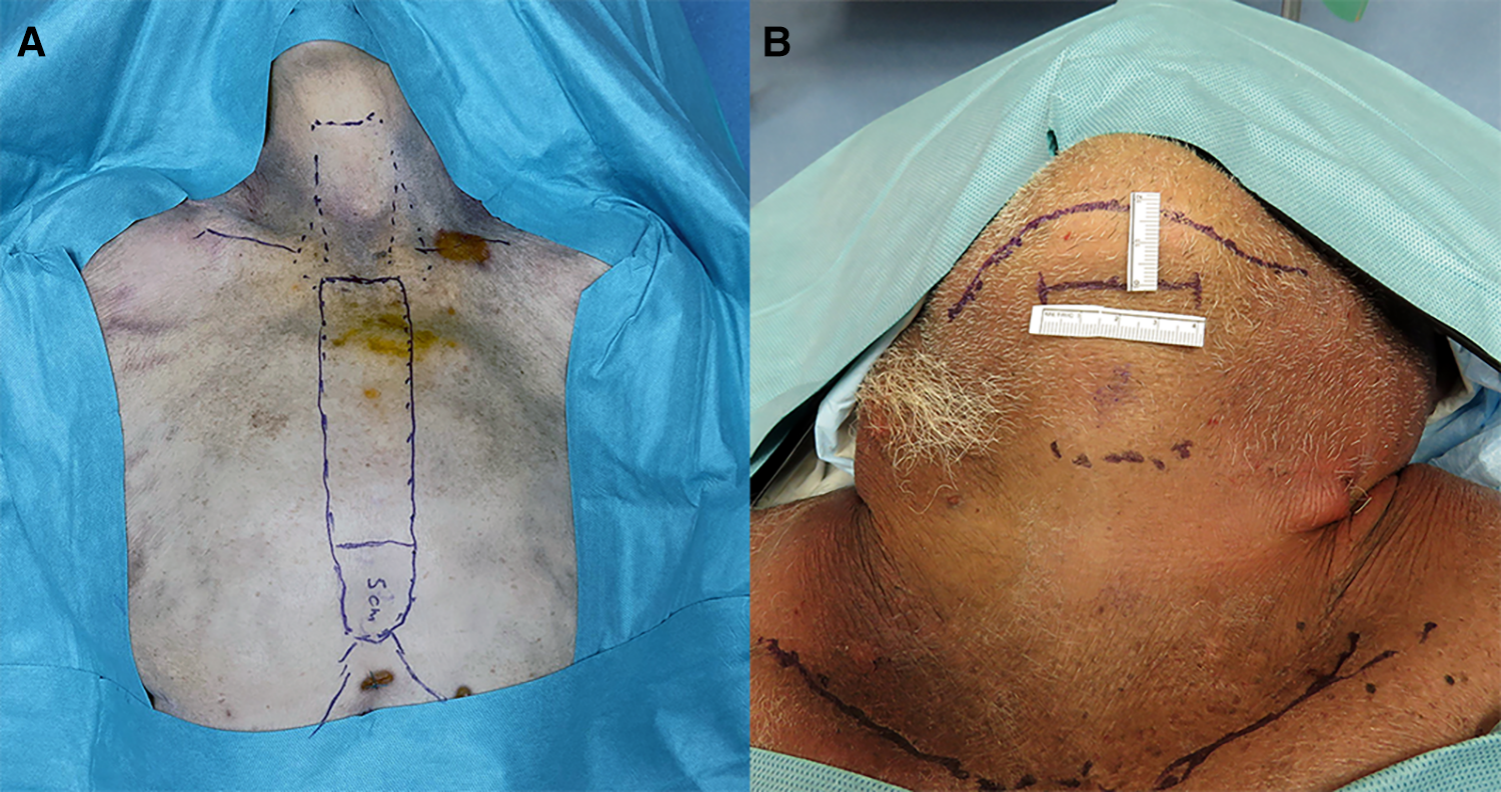
(**A**) Presternal approach: delineation of the dissection area and incision site 5 cm from the tip of the xiphoid process in cadaver 2. (**B**) Submental approach: incision site marking at 2 cm below the mandible in cadaver 5.

**Figure 2:**
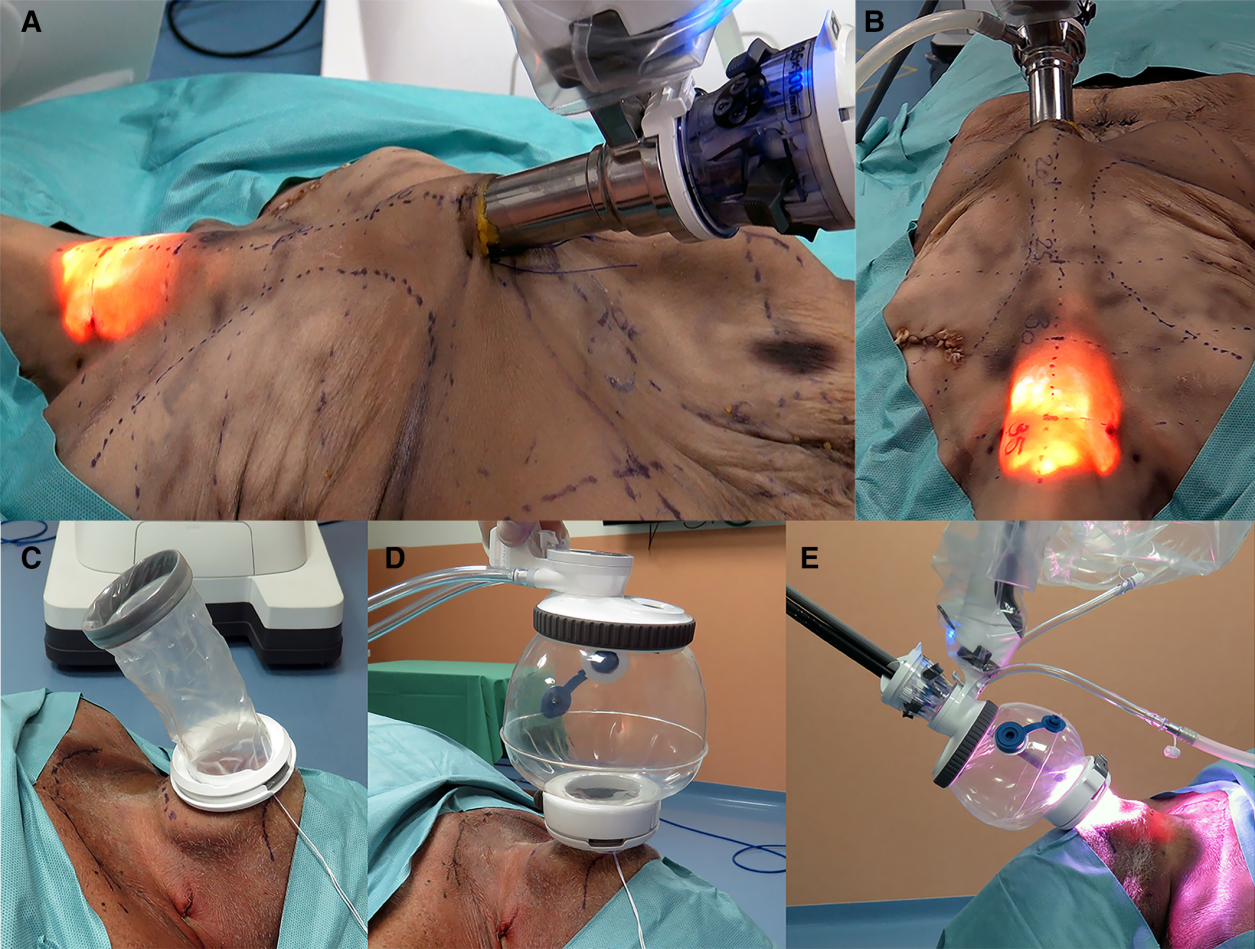
(**A,B**) Presternal approach: SP system docked to the cannula at the presternal incision approaching the model from the left. (**C–E**) Submental approach: (**C**) wound retractor placed in the incision, (**D**) SP access port attached and insufflated, and (**E**) SP system docked submental.

**Figure 3:**
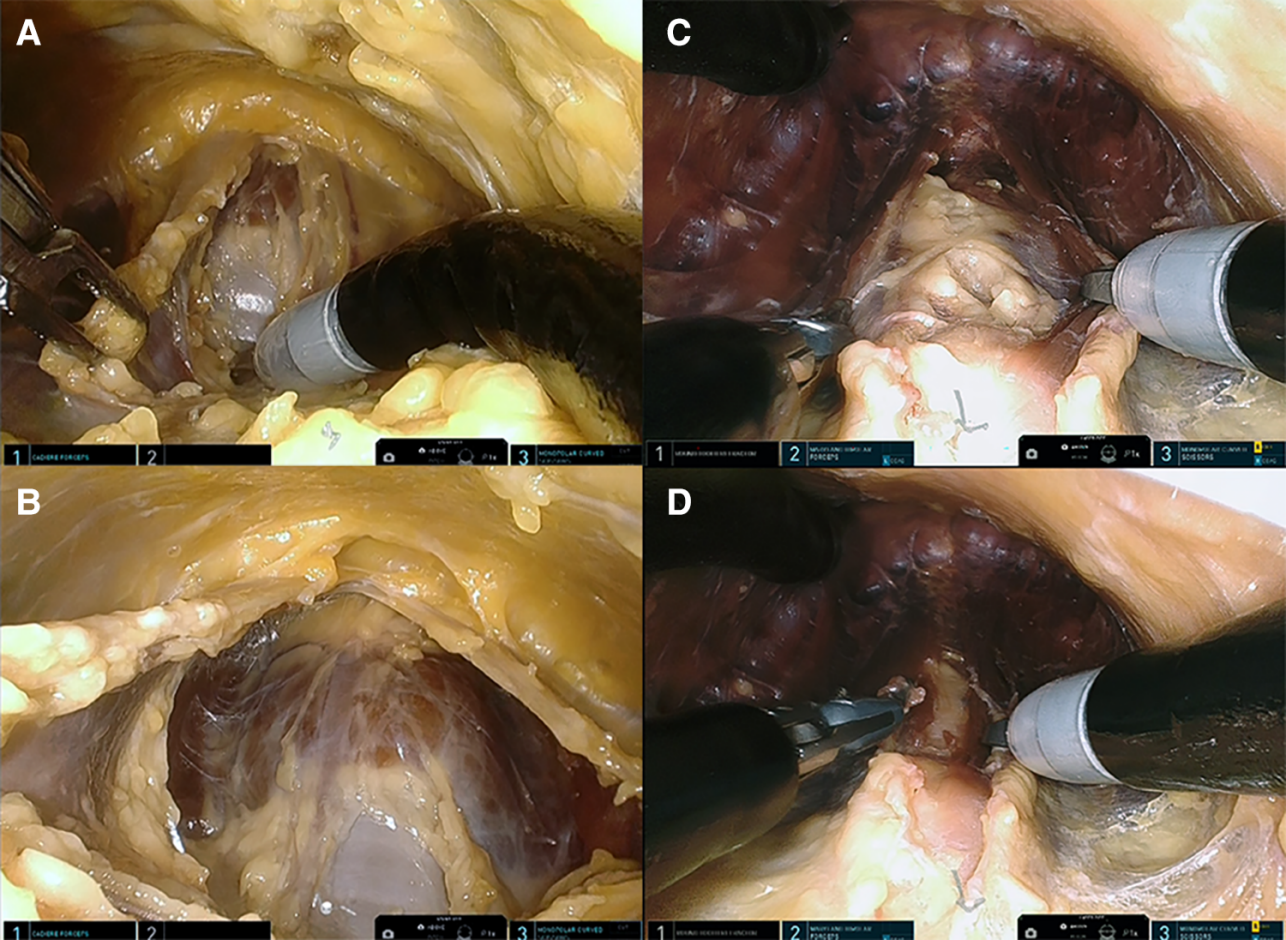
(**A**) Presternal approach: midline separation of the strap muscle with trachea coming into view (cadaver 1). (**B**) Presternal approach: anterior surface of the trachea and isthmus are visualized (cadaver 1). (**C**) Submental approach: separated strap muscle with trachea behind it (cadaver 5). (**D**) Submental approach: anterior surface of the trachea and isthmus are visualized (cadaver 5).

**Figure 4:**
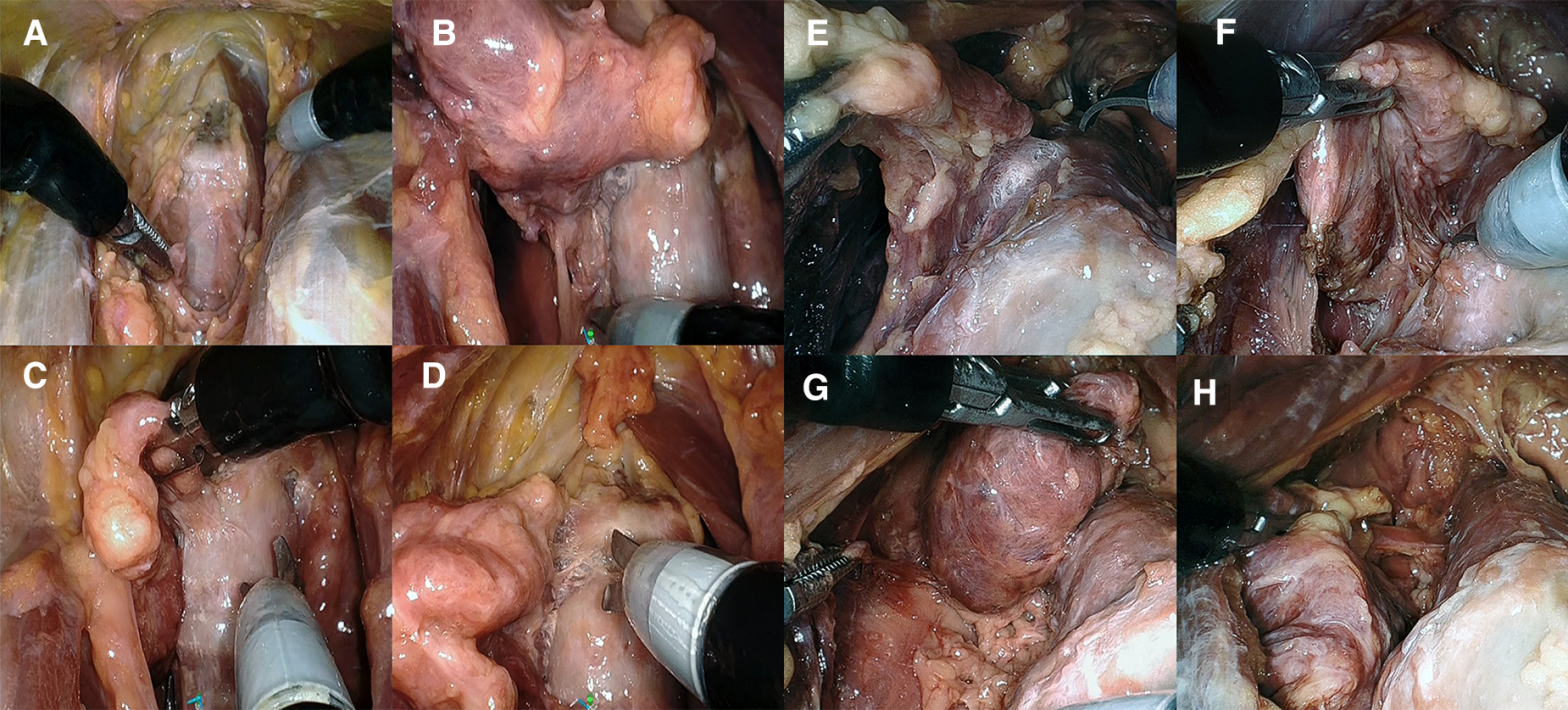
(**A–D**) Presternal approach in cadaver 2: (**A**) completed division of isthmus, (**B**) right posterolateral aspect released to access the recurrent laryngeal nerve, and (**C**, **D**) transection of the right ligament of Berry in the posteromedial and anteromedial plane. (**E–H**) Submental approach in cadaver 5: (**E**) transection of the left ligament of Berry and (**F**) right posterolateral aspect released to access the recurrent laryngeal nerve. (**G**) Superior pole and thyroid body released from trachea. (**H**) Final inferior attachment prior to transaction freeing the right lobe.

**Figure 5:**
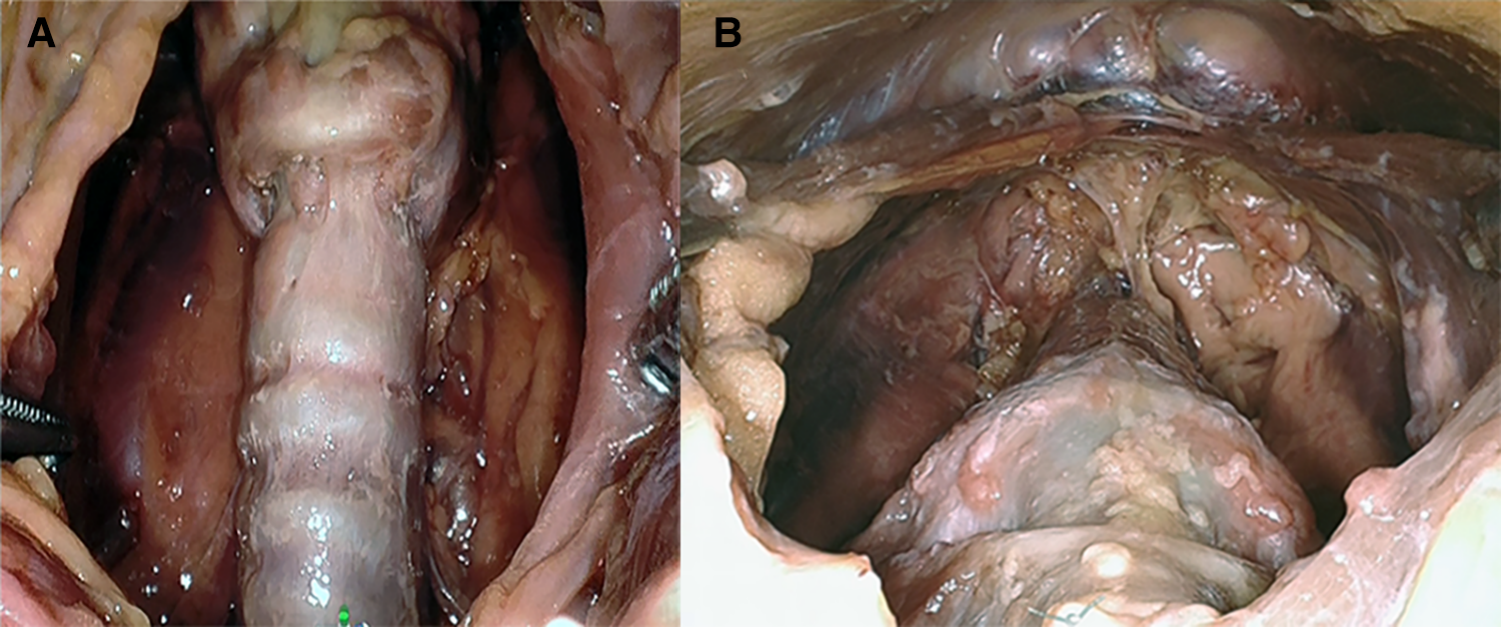
**(A)** Presternal approach: surgical view of the resected area after removal of the specimen in cadaver 2. **(B)** Submental approach: surgical view of the resected area after removal of the specimen in cadaver 5.

**Figure 6:**
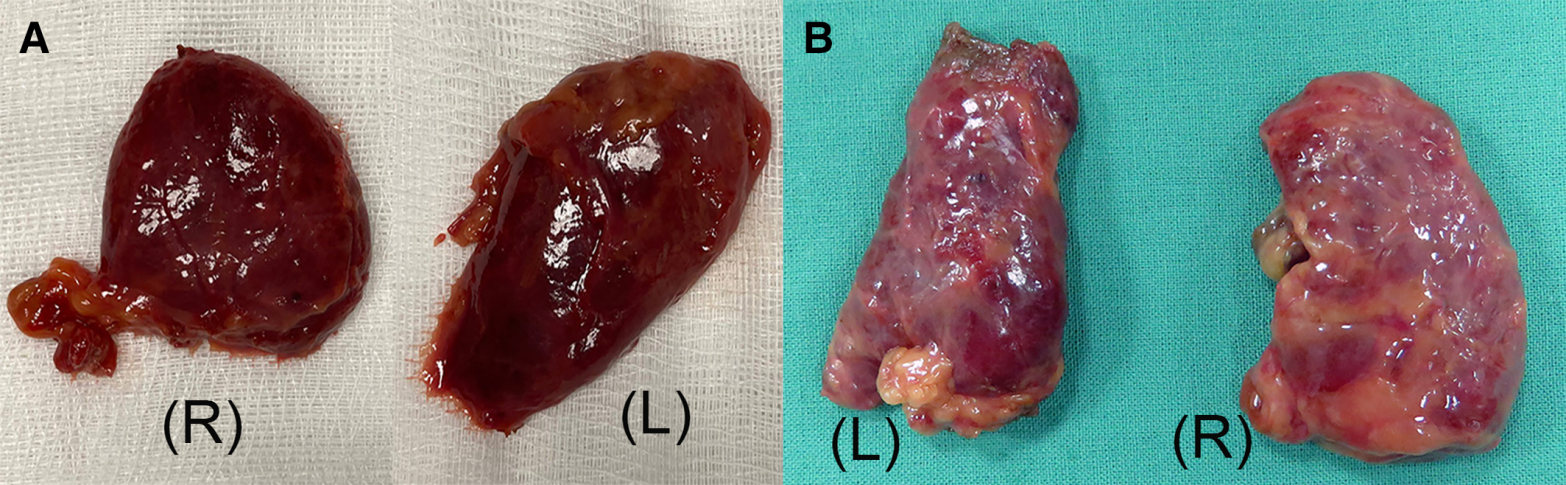
**(A)** Presternal approach: the right (R) and left (L) thyroid lobe specimen with capsule intact from cadaver 2. (**B**) Submental approach: the right (R) and left (L) thyroid lobe specimen from cadaver 5.

The procedures were performed similarly to the technique described by Lee et al. (3). Cadaver models 1 and 2 were placed in the supine position with the neck extended. A 3-cm skin incision was created at about 5 cm from the tip of the xiphoid process slightly above the level of the inframammary fold (IMF) on top of the sternum, and the presternal dissection area was delineated with a marking pen and measured (Figure 1A). A subcutaneous tunnel was bluntly dissected with long Metzenbaum scissors up to the inferior border of the suprasternal notch. The SP obturator was introduced next to deepen and widen the tunnel sufficiently for the SP cannula to be placed. The SP cannula was inserted directly through the skin incision into the presternal tunnel. A purse-string suture was applied around the SP cannula to avoid slipping since skin tonus was not existing in the models. Next, CO_2_ insufflation was established at 5 mmHg, opening up the workspace, and kept low to avoid subcutaneous emphysema. The SP system patient cart was docked to the cannula approaching the patient from the left (Figures 2A,B). Monopolar curved scissors were installed on the right, a Cadiere forceps on the left, and the endoscope in the middle in the “camera above” configuration. After minor dissection, the suprasternal notch was reached, and the sternocleidomastoid muscles on the left and right came into view. The flap was developed up to the thyroid cartilage. Next, a midline incision through the superficial layer opening the cervical fascia between the strap muscles was performed. The sternohyoid was separated with the monopolar curved scissors, and dissection was extended until the anterior surface of the trachea, the cricoid cartilage, and the isthmus were fully visualized (Figures 3A,B). The isthmus was divided, and the thyroid gland retracted medially to separate the thyroid capsule off the strap muscle on both sides (Figure 4A). In cadaver 1, surgery was terminated at this point in the procedure as this initial lab merely aimed to assess sufficient exposure and access to the thyroid gland with the presternal SP approach. In cadaver 2, surgery continued on the right lobe after adding a fenestrated bipolar forceps on Arm 2 of the SP to aid in exposure for the preservation of critical structures (laryngeal nerve, etc.) and dissection of the upper thyroid pole. This instrument in Arm 2 could be switched between left-hand and right-hand control to facilitate optimal traction on the tissues depending on the operator's needs. Dissection was continued laterally to fully release the posterolateral side of the thyroid gland and get access to the recurrent laryngeal nerve (Figure 4B). The plane superficial to the nerve was followed up to the ligament of Berry, which was then transected (Figure 4C). Attention was then shifted to the upper thyroid pole, releasing the remaining attachments to the strap muscle laterally. The superior thyroidal vessels were divided with bipolar and monopolar cautery energy and opened up the plane between the cricothyroid muscle and the thyroid gland. Residual connections along the ligament of Berry were cut, and the right lobe was completely freed (Figure 4D). The left lobe was dissected in a similar fashion while changing the fenestrated bipolar forceps and monopolar curved scissors on the SP arms per procedural needs. Figure 5A shows the surgical view of the resected area, and Figure 6A shows both thyroid lobes with the thyroid capsules intact. Both lobes were removed through the presternal tunnel after completion of the resection, and the SP cannula was undocked. Adjustments of the EGM were not required during the procedure since the thyroid gland, as the surgical target, was situated exactly midline in a straight line from the port.

### Submental thyroidectomy

The procedures were performed similar to the steps described by Kim et al, (10). Cadaveric models 3, 4, and 5 were placed in a supine position with the neck overstretched by a saline bag under the shoulders. In cadaver 3, a submental incision 3 cm in length was made at about 1 cm distance to the mandible (Figure 1B). Next, the skin flap was developed in the subcutaneous plane inferiorly toward the thyroid and the small SP access port was placed once sufficient flap developed. CO_2_ insufflation was initiated at 6 mmHg, and the SP system was docked to the access port (Figures 2C–E). The endoscope was installed in the “camera below” configuration, with initially only a fenestrated bipolar forceps in the left and monopolar curved scissors in the right hand. The skin flap was further developed down to the sternal notch, and the strap muscles were separated (Figures 3C,D). At this point, the endoscope was moved to the “camera above” configuration and an additional Cadiere forceps was inserted to aid in retraction. After dividing the isthmus, left lobectomy was started by retracting the sternohyoid muscle and releasing the thyroid beginning at the superior pole. The dissection progressed until the superior parathyroid gland was identified and released. Next, the ligament of Berry was dissected to free the superior part completely. The dissection continued inferiorly along the right laryngeal nerve (RLN) and the lower parathyroid gland was identified (Figures 4E,F). The same steps were followed to perform the right lobectomy for a total thyroidectomy (Figures 4G,H). The specimen was placed into the SP access port chamber for subsequent removal.

We had some difficulties in seating the SP access port in the first submental procedure due to laxity in the neck skin and some fibrotic tissue on the anterior aspect of the mandible. This caused the wound retractor to create bruising around the soft tissues of the inferior aspect of the jaw and around the area of the mental foramen. Therefore, we changed the approach for the procedures in cadavers 4 and 5. A 4-cm incision was made 2 cm below the edge of the chin. To improve skin flap creation and exposure, we elevated the neck skin with six external sutures placed along the midline of the neck at a 3–4 cm distance from each other. Also, the wound retractor on the SP access port was not rolled all the way down, leaving enough slack to allow for stretching around the mandible. Thyroidectomies in cadavers 4 and 5 were done in the same fashion as in cadaver 3.

## Results

### Presternal thyroidectomy

In cadavers 1 and 2, creating the skin flap and docking of the SP system took 6 min in each case.

The initial exposure of the thyroid gland after separation of the strap muscles was performed within an acceptable time (29 min for cadaver 1, 30 min for cadaver 2) in a single-incision presternal approach (Table 2). Having convinced ourselves that sufficient exposure and access with the presternal SP approach was provided in cadaver 1, we pursued developing this technique in a subsequent lab with cadaver 2. In this case, we continued with isthmectomy and the resection of both thyroid lobes, which took an additional 51 min of surgery time, totaling 83 min to complete the bilateral thyroidectomy. Figure 5A shows the surgical view of the resected area, and Figure 6A shows both thyroid lobes with the thyroid capsules intact. No adjustments of the SP EGM were required throughout the procedure to keep the trajectory of the EGM aligned with the target tissue.

**Table 2:** Results.

Procedure step^a^	Cadaver 1	Cadaver 2	Cadaver 3	Cadaver 4	Cadaver 5
Approach	PS	PS	SM	SM	SM
Setup—docking time^b^	6	6	5	8	7
Time to start the robotic part	10	11	10	12	15
Time to thyroid exposure	29	30	27	27	19
Time to isthmectomy	—	32	34	35	23
Total resection time^c^	—	83	83	121	67

PS, presternal; SM, submental.

^a^ All times are listed in minutes and are counted from the initial skin incision and accumulative.

^b^ Time from first skin incision until SP system was docked and instruments engaged, includes skin flap development.

^c^ Resection time for left and right lobe together.

### Submental thyroidectomy

In cadavers 3, 4, and 5, creating the skin flap and docking of the SP system took between 5 and 8 min (Table 2). Initial exposure of the thyroid gland was achieved in 27 min in cadavers 3 and 4 and in 19 min in cadaver 5. Completing the total thyroidectomies in cadavers 3, 4, and 5 took 83, 121, and 67 min, respectively. The surgical view of the resected area is shown in Figure 5B, while Figure 6B shows both thyroid lobes with the thyroid capsules intact. In cadavers 4 and 5, we did not see any interference of the wound retractor with the inferior aspect of the jaw and soft tissues since we kept the sleeve of the SPO access port longer to enable motion around the mandible*.* Large adjustments of the EGM were not required during these procedures, and any adaption to surgical view was easily controlled from the surgeon's console.

## Discussion

Performing remote-access thyroidectomy through either a presternal or submental approach with the da Vinci SP system was feasible in our cadaver study. We believe that these approaches might address some of the concerns of robotic remote-access thyroidectomy and have some particular advantages in consideration to other robotic thyroidectomy methods. No additional ports or retractor devices were required to expose the gland and complete the bilateral resection. The ability of the SP system to work in a small narrow space decreases the amount of skin flap that needs to be dissected for access to the gland. Specifically, in the presternal approach, there is a limited risk of tissue damage during the creation of the presternal flap as the posterior surface is a bone and no nerves or breast tissue lie subcutaneously in this empty space. Dissecting the presternal tunnel or submental flap required only a small skin area and compares well with the mean area of dissection required for FL and is better than for TA, which Singer et al. (10) reported in their morphometric analysis. The distance from the incision site to the thyroid gland, with an average of 15 cm in all cadavers, is like BABA, TA, and FL and shorter than what was reported in TORT (19 cm) (11). Since only a single arm needs to be docked with the SP system, complexity is reduced and the setup was very quick, shortening the procedure time. Comparing our combined skin flap development and docking times from these initial labs with docking times to earlier patient series in other robotic approaches shows a noteworthy reduction in the required time. It took us between 5 and 8 min in the models, which is much shorter than what has been reported in multiport approaches for TA (28 min) (1), FL (68 min) (12), and BABA (61.8 min) (2) and is also shorter than the 38 min reported by Kim et al. (9) for the SP system in their series. However, our procedures were performed on deceased patients, and we did not have to deal with hemostasis and other surgical matters that would apply to a live patient. Yet, we believe that for a trained surgical team in a clinical case, it would be possible to get close to these times after some experience. Due to the midline exposure similar to open surgery and familiar to thyroid surgeons, a wide surgical view of the gland in a natural position is enabled. This allows for total thyroidectomy approaching both lobes. The parallel configuration of the instruments through the SP cannula enables using all three SP instruments simultaneously for exposure with full dexterity. Also, since the patient is placed supine and the neck stays straight throughout the surgery, there would be no movement or shifting of the intraoperative nerve monitoring (IONM) tube, and the risk of brachial plexus injury due to arm positioning would be eliminated.

Potential disadvantages of our method might be the possibility of hypertrophic scarring at the incision site for the presternal approach. In the submental approach, the scar can be hidden cosmetically, like with a facelift procedure. As we performed the procedures under CO_2_ insufflation, any complications related to it (subcutaneous emphysema, embolism, etc.) would apply in clinical cases. Another concern is the unavailability of an ultrasonic dissector adapted for the SP system like the Harmonic ACE (Johnson & Johnson Medical Inc., New Brunswick, NJ, United States), which is the preferred dissection instrument by many robotic thyroid surgeons. However, in their initial SP thyroidectomy series of 10 patients, Kim et al. (9) became comfortable after the third case with the mono- and bipolar SP instrumentation energized by the integrated electrosurgical unit and found it a safe and effective alternative to the harmonic scalpel.

Our feasibility study has several shortcomings because it was performed in cadavers. First, any postoperative outcomes such as pain, voice impairment, swallowing, complication rates, or the impact of ventilation and bleeding from structures in a living human were not possible. A major concern remains satisfactory postoperative cosmesis with the presternal approach and the incision placed right between inframammary folds. While the incision might be hidden in a woman at the position where the bridge between the left and right cup of a bra would sit, it would be visible on the bare chest in a male. The question remains if this is an acceptable result. Cao et al. (13) performed endoscopic thyroidectomy with a 15-mm port placed in the same presternal location as in our study, and of 282 patients, 78.7% rated the cosmetic result as extremely satisfactory and an additional 13.9% rated as good, despite the presternal 15-mm incision. Nevertheless, the cosmetic benefit is something to be evaluated. In the submental approach, the interference with the wound retractor was an issue in the first cadaver but was resolved by moving the incision slightly inferior and keeping some slack in the sleeve of the access port in the subsequent cases. Nevertheless, in cadaver 4, the longer sleeve was punctured by the monopolar scissors during an instrument change, leading to some loss in CO_2_ insufflation. Care must be taken at the access port interface during these maneuvers to avoid any issues. Hence, unless these approaches are investigated in clinical cases, we will not be able to answer whether they will provide any significant patient benefit in the postoperative outcome, including a reduction in cost due to shorter operating room (OR) time while at the same time not ignoring oncologic outcome with these new surgical techniques. This also applies to a comparison with laparoscopic surgery. Our experimental cadaveric setup was not designed to determine whether a meaningful outcome improvement is achievable compared to laparoscopy. We can only imagine that a submental or presternal approach with laparoscopic instrumentation would be challenging to execute. Our study did not assess specific challenges that surgeons with limited experience with the da Vinci SP system might encounter in performing the described techniques. Lee et al. (14) compared two patient cohorts in retroperitoneal adrenalectomy treated with either the da Vinci SP or a da Vinci Si/Xi (multiport) system. All surgeries were done by the same surgeon (SWK, co-author of this report) with more than 10 years of experience with the multiport da Vinci systems. They found no difference in the mean operation time on these first da Vinci SP cases compared to the da Vinci Si/Xi cases from their previous experience. The team was able to reduce the OR time reasonably in the starting stage and concluded that the operation times with da Vinci SP would likely be shorter as future experiences are accumulated and that SP facilitated easier and more convenient operations for surgeons. Therefore, we believe that the SP learning curve is relatively short and not an obstacle for experienced robotic surgeons.

This study demonstrates the feasibility of a bilateral thyroidectomy with the da Vinci SP system through single-incision presternal and submental approaches in a cadaver model. While our report does not claim to give a comprehensive picture in comparison to the other robotic thyroidectomy approaches, it suggests that these presternal and submental single-port thyroidectomies compare promisingly with other currently applied robotic methods. The amount of skin flap development required for these approaches was minimal, and docking of the SP system was quick. No additional ports were required to expose the gland and complete the bilateral resection. Further studies will be needed to assess whether a presternal or submental thyroidectomy with the da Vinci SP system yields true clinical benefits in real patients.

## Data Availability

The original contributions presented in the study are included in the article, further inquiries can be directed to the corresponding author.
